# Biodegradable Packaging from Agricultural Wastes: A Comprehensive Review of Processing Techniques, Material Properties, and Future Prospects

**DOI:** 10.3390/polym17162224

**Published:** 2025-08-15

**Authors:** Bekzhan D. Kossalbayev, Ayaz M. Belkozhayev, Arman Abaildayev, Danara K. Kadirshe, Kuanysh T. Tastambek, Akaidar Kurmanbek, Gaukhar Toleutay

**Affiliations:** 1 Department of Chemical and Biochemical Engineering, Geology and Oil-Gas Business Institute Named After K. Turyssov, Satbayev University, Almaty 050043, Kazakhstan; 2 Sustainability of Ecology and Bioresources, Al-Farabi Kazakh National University, Al-Farabi Ave. 71, Almaty 050040, Kazakhstan; tastambeku@gmail.com (K.T.T.); kurmanbek.akaidar@mail.ru (A.K.); 3 International Faculty, Asfendiyarov Kazakh National Medical University, Almaty 050012, Kazakhstan; 4 Department of Chemistry, University of Tennessee, Knoxville, TN 37996, USA

**Keywords:** plant waste, sustainable packaging, lignocellulosic biopolymers, circular bioeconomy, pulp molding

## Abstract

Packaging demand currently exceeds 144 Mt per year, of which >90% is conventional plastic, generating over 100 Mt of waste and 1.8 Gt CO_2_-eq emissions annually. In this review, we systematically survey three classes of lignocellulosic feedstocks, agricultural residues, fruit and vegetable by-products, and forestry wastes, with respect to their physicochemical composition (cellulose crystallinity, hemicellulose ratio, and lignin content) and key processing pathways. We then examine fabrication routes (solvent casting, extrusion, and compression molding) and quantify how compositional variables translate into film performance: tensile strength, elongation at break (4–10%), water vapor transmission rate, thermal stability, and biodegradation kinetics. Highlighted case studies include the reinforcement of poly(vinyl alcohol) (PVA) with 7 wt% oxidized nanocellulose, yielding a >90% increase in tensile strength and a 50% reduction in water vapor transmission rate (WVTR), as well as pilot-scale extrusion of rice straw/polylactic acid (PLA) blends. We also assess techno-economic metrics and life-cycle impacts. Finally, we identify four priority research directions: harmonizing pretreatment protocols to reduce batch variability, scaling up nanocellulose extraction and film casting, improving marine-environment biodegradation, and integrating circular economy supply chains through regional collaboration and policy frameworks.

## 1. Introduction

Currently, global plastic production exceeds 360 million tons annually, with packaging representing nearly 40% of this total [1]. However, less than 10% of plastic packaging is effectively recycled, while the rest accumulates in landfills or disperses into the environment where it degrades into persistent microplastics. Ingestion of microplastics (<5 mm) by marine organisms has been detected in >80% of species, impairing feeding and reproduction, and human dietary exposure can exceed 50,000 particles per year, with documented inflammatory responses upon ingestion [2,3]. The incineration of plastic waste also significantly contributes to greenhouse gas emissions and the release of toxic pollutants [3]. Directives, such as EU 2019/904, mandate a 50% reduction in specific single-use plastic items by 2025, driving a ~20% annual increase in global bioplastic production capacity. At the same time, the agricultural and food processing sectors annually generate abundant lignocellulosic residues, including straw, husks, stalks, peels, and pomace [4]. These residues are rich in naturally biodegradable polymers such as cellulose, hemicellulose, lignin, and pectin, which can be converted into films, fibers, and composites [5]. Repurposing 1 Mt of rice straw can avoid 2500 t CO_2_-eq from open burning annually and replace 500 t of virgin polymer, cutting raw-material costs by ~25 % (USD 900 vs. USD 1200 per t) when integrated into existing compounding processes [4].

Three principal strategies have been explored to convert plant waste suitable viable packaging materials: valorization of various wastes through mild pre-treatments (alkaline, enzymatic, or using deep-eutectic solvents) to isolate functional polymer fractions for producing fibers, powders, or purified biopolymers [5], mechanical and chemical processing methods such as pulp molding, hot pressing, extrusion, crosslinking, and plasticization to fabricate structurally robust and water-resistant packaging formats including trays, films, and foams [6], and composite formulations, wherein waste-derived components are blended with other biopolymers such as starch, polylactic acid (PLA), polyhydroxyalkanoates (PHA), or chitosan to optimize mechanical strength, barrier properties, and cost-effectiveness for varied packaging needs [7].

**Figure 1:**
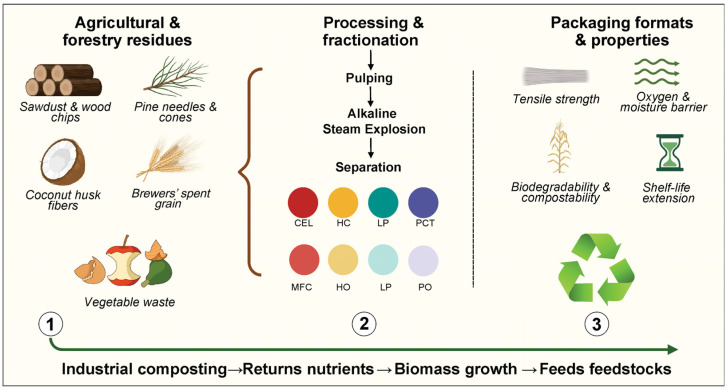
Agricultural and forestry waste conversion into functional packaging materials: Note: (1) sources; (2) processing pathways; (3) integration into the circular bioeconomy. CEL, cellulose; HC, hemicellulose; LP, lignin; PCT, pectin; MFC, microfibrillated cellulose; HO, hemi-oligosaccharides; LP, lignin phenolics; PO, pectic oligosaccharides. Created with BioRender.com (License No. FO28F9LHV7).

In the recent literature, several concise reviews have examined specific agricultural residues [8,9], such as cereal straws [10], nut shells [11], and fruit pomaces [12], focusing primarily on compositional analyses and singular processing techniques [13,14]. However, these treatment methods typically overlook a comprehensive comparison of the physicochemical characteristics of different feedstocks, mechanistic frameworks linking biopolymer structure to functional properties, and a decision-making framework for selecting waste streams based on regional availability and target application requirements. Our review fills these gaps by systematically mapping major crop residues, providing detailed reaction pathway diagrams correlated with end-use behavior, and offering a practical matrix for feedstock selection (Figure 1).

## 2. Sources of Plant Waste

As mentioned earlier, plant-based residues arise at all stages of agriculture, food processing, and forestry operations, including materials such as cereal straw, husks, fruit peels, and other industrial by-products. Each of these materials has a unique biochemical composition, with variable ratios of cellulose, hemicellulose, lignin, pectin, and minor extractives, which influences both processing behavior and the performance characteristics of the final packaging material. Therefore, classifying these wastes by origin and composition is essential for selecting the right pretreatment and fabrication strategies.

Table 1 summarizes various plant-based waste sources, their typical composition, examples of how they are used in sustainable packaging, and the key benefits or properties of the resulting materials.

**Figure 2:**
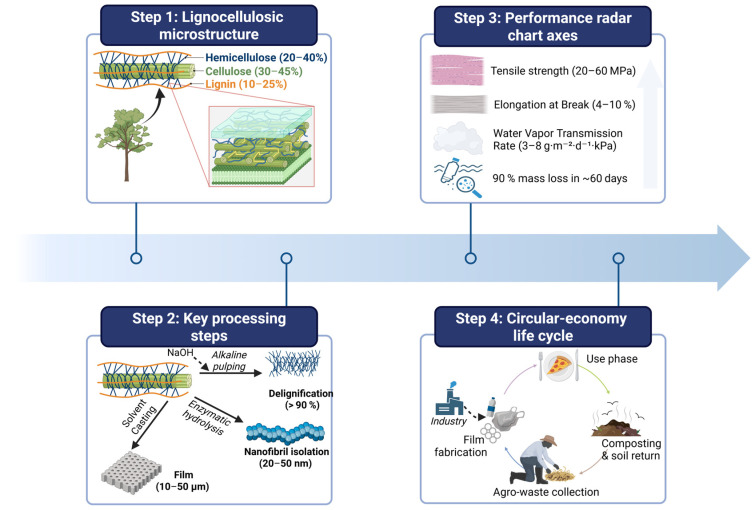
Overview of agro-waste to biodegradable film pathways. Created with BioRender.com (License No. NU28KK11OC).

Following the compositional summary in Table 1, Figure 2 integrates four complementary views of lignocellulosic feedstock conversion: (a) the native plant cell wall architecture, in which crystalline cellulose microfibrils, amorphous hemicellulose and hydrophobic lignin domains collectively determine mechanical strength, moisture sensitivity and thermal resistance, (b) the sequential processing pathway, alkaline pulping for delignification, enzymatic hydrolysis to isolate nanofibrils, casting or extrusion, that transforms raw residues into uniform films, (c) a plot of key performance metrics, and (d) the circular-economy loop, illustrating field-residue collection, film fabrication, single-use application and >90% mass recovery via industrial or home composting.

### 2.1. Agricultural Residues

Molded pulp trays made from waste wheat straw fibers have demonstrated mechanical properties suitable for many packaging applications. A molded pulp tray composed of 80 wt% straw fiber, with the fibers held together by a biodegradable starch-based adhesive matrix, exhibited a tensile modulus of approximately 0.47 MPa. While this modulus is lower than that of many solid plastics, it reflects the compliance of the highly porous fiber-binder network and remains fully biodegradable [34].

Rice husks have also been effectively incorporated into bioplastic films and composites. For example, starch-based films reinforced with rice husk fibers showed enhanced tensile strength and reduced reliance on synthetic polymers [35]. Numerous studies have evaluated thermoplastic composites incorporating rice husk fillers for packaging applications, underscoring their viability and performance [20,21].

Beyond rice and wheat residues, other crop by-products are also gaining attention. Sugarcane bagasse, a fibrous residue from sugar extraction, is already utilized in commercial biodegradable tableware. Several studies report that, when properly processed to minimize voids and with appropriate fiber–matrix adhesion, bagasse-reinforced starch or PLA composites can exhibit enhanced stiffness. Under these optimized, essentially void-free conditions, the tortuous diffusion path created by well-dispersed fibers may also lower water vapor transmission rates compared to the neat polymer, although overall water uptake can still increase depending on fiber loading and treatment [18]. A recent review confirmed that, when manufactured via compression molding with compatibilizers, these bagasse, PLA composites achieve competitive mechanical properties alongside full composability [19].

Corn residues, such as husks and stalks, have also been studied for their packaging potential. One of the investigations revealed that maize husk fibers could replace up to 80% of conventional fibers in paper packaging without compromising mechanical integrity [20].

Agricultural residues represent a highly attractive feedstock for sustainable packaging due to their abundance and rich cellulose content. By converting straw, husks, and stalks into functional packaging, significant environmental benefits can be realized, including reduced open burning and the promotion of eco-friendly alternatives [17].

### 2.2. Fruit and Vegetable Processing By-Products

The food processing industry produces considerable amounts of plant waste, including peels, skins, pomace, and seed hulls. These residues are typically rich in pectin, fibers, and bioactive compounds, offering valuable potential for the development of edible or bioactive packaging materials. Fruit peels, in particular, have been successfully utilized to fabricate bioplastic films. For instance, Zhang et al. (2024) utilized the intrinsic components of mixed fruit peel waste to develop a biodegradable, antimicrobial bioplastic film capable of extending the shelf life of perishable fruits, owing to its antioxidant properties [22].

In a similar vein, Kharb and Saharan (2025) produced an antibacterial food packaging film derived from mixed fruit peels, showing that peel extracts can effectively impart antimicrobial properties to biodegradable films [23]. Apple pomace, a residue from juice and cider production, is another significant resource, abundant in pectin and polyphenols. These components have been incorporated into active packaging systems; for example, Varghese et al. (2023) integrated apple peel polyphenols into chitosan films, creating antimicrobial packaging suitable for meat products [17].

Citrus processing waste, particularly peel, is a notable source of pectin and flavonoids. Citrus-derived pectin has been used both as a blend component in film formulations and as a functional coating to enhance barrier properties of paper-based packaging. Kasaai and Moosavi (2017) [24] isolated hydrophobic fractions from mandarin (*Citrus reticulata*) peel and leaves via solvent extraction and characterized them by gas chromatography–mass spectrometry and infrared spectroscopy. These terpene-rich extracts were emulsified with a cellulose nanofibril binder to form an aqueous coating formulation, which was applied to food-grade Kraft paper by bar coating and then dried. The resulting coating both filled the paper’s pore network and formed a continuous hydrophobic layer, leading to significant reductions in water vapor permeability and air transmission rate, as well as enhanced antioxidant activity, demonstrating its promise for food packaging applications.

Vegetable by-products, including potato peels, carrot pomace, and tomato skins, are also being valorized. Potato peel starch and fibers have been used to produce edible films and loose-fill packaging foams [25]. Cruz-Tirado et al. (2019) enhanced a sweet potato starch-based foam with fibers from asparagus peel and sugarcane bagasse, yielding a compostable foam tray with improved mechanical strength and reduced water absorption [27].

Moreover, Tran et al. (2022) demonstrated the viability of vegetable residues such as dried parsley and spinach stems, which were micronized and incorporated into a polymer matrix via hot pressing, acting as effective functional fillers [28].

**Figure 3:**
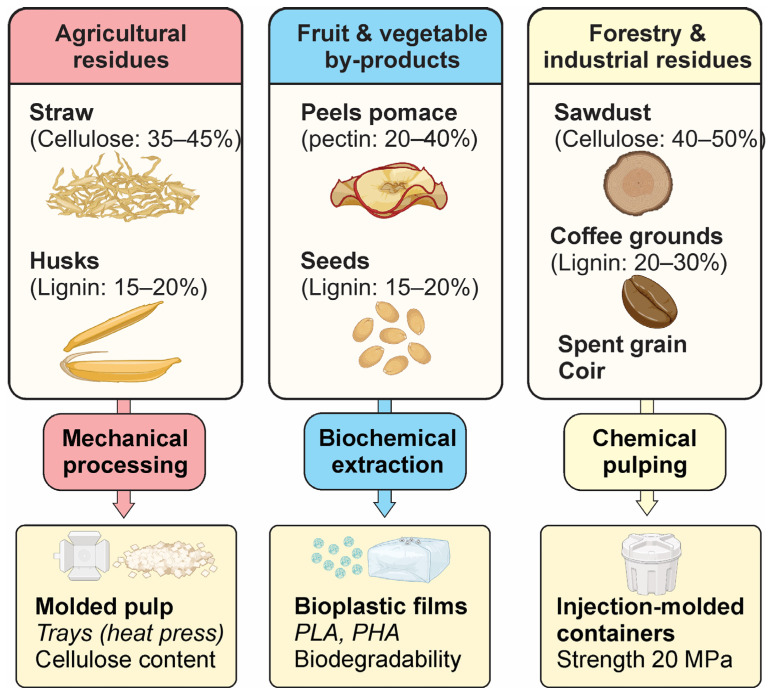
Sources of plant waste and their packaging applications. Created with BioRender.com (License No. IY28F9LSP2).

Fruit and vegetable processing by-products offer a promising secondary application as raw materials for packaging [36]. Their inherent biopolymers, like pectin and vibrant phytochemicals, enable the development of active and intelligent packaging solutions. A conceptual diagram (Figure 3) illustrates the valorization pathway, highlighting the transformation of materials such as orange peels and apple pomace into bioplastic films or functional coatings.

### 2.3. Forestry and Industrial Plant Residues

Brewers’ spent grain (BSG), a by-product of the beer brewing process comprising cellulose, protein, and lignin, is another promising material. Produced in vast quantities globally, BSG has been integrated into biopolymer matrices to produce packaging films, simultaneously addressing waste management issues and providing a cost-effective raw material [31].

Waste coffee grounds, another abundant food industry residue typically disposed of in landfills, have also been converted into packaging materials. Through solvent pre-treatment to remove residual oils and liberate cellulose, the material was pulped and processed into biodegradable paper. This paper could incorporate bioactive plant compounds, imparting antioxidant and antimicrobial functionalities.

Forestry activities and plant fiber processing industries generate significant waste streams, sawdust and wood chips from lumber production, pine needles and cones from forest management, coconut husk fibers, and agro-industrial by-products, such as residual brewers’ grain and coffee grounds, that can be repurposed into sustainable packaging applications. These lignocellulosic residues are typically rich in cellulose, hemicellulose, lignin, and phenolic extractives, and undergo tailored pre-treatments, such as alkaline pulping, enzymatic hydrolysis, or steam explosion, to isolate functional biopolymer fractions for composite formation and moldable pulp production.

Sawdust and wood chips can be mechanically refined and pulp molded into trays and panels with tensile strengths comparable to expanded polystyrene, while pine residues have been valorized into microfibrillated cellulose-based paper with ethylene-scavenging properties, extending fresh produce shelf life. Coconut coir fibers, noted for their high lignin content and robust fibrous network, reinforce starch- or PLA-based films, boosting both tensile strength and moisture resistance.

Agro-industrial wastes such as brewers’ spent grain and spent coffee grounds serve dual functions: as structural fillers and as sources of antioxidants or antimicrobial compounds, thereby adding active functionality to packaging films and further valorizing by-products. The abundant lignin and other underutilized components in these residues support a zero-waste, circular bioeconomy by transforming industrial by-products into high-performance, biodegradable packaging materials.

Kumar et al. (2023) demonstrated the valorization of pine needle biomass, a forest residue often associated with fire risk, into paper-based active packaging [29]. Pine needle pulp was first blended with micro-fibrillated cellulose to form a cohesive fiber network, then functional additives, namely 20–30 wt% halloysite nanotubes (HNTs), tubular aluminosilicate clays with high surface area and exchangeable sites, were incorporated to impart ethylene’s scavenging capacity. The resulting composite paper exhibited an ethylene scavenging efficiency of approximately 62% and extended the shelf life of fresh bananas by up to five days under ambient storage conditions, offering a sustainable, circular-economy alternative to conventional plastic films.

Other valuable sources include residues from the pulp and paper industries, which can be molded into packaging formats, as well as waste fibers from the textile sector and natural fiber crops. For instance, Nasri et al. (2023) reported that incorporating flax fiber residues into polypropylene significantly enhanced the mechanical strength of the composite due to flax’s high cellulose content [33].

Forestry and industrial plant residues represent a supplementary feedstock stream for eco-friendly packaging development. Although these materials often contain high levels of lignin or extractives, which may necessitate specific pre-treatments, their use supports a zero-waste approach by converting industrial by-products into high-value packaging while alleviating disposal challenges [37].

## 3. Chemical Composition of Lignocellulosic Materials

Unlocking the full potential of lignocellulosic residues requires tailored pretreatment and conversion platforms that overcome biomass recalcitrance and yield high-purity intermediates. From physicochemical treatments, each approach balances delignification, hemicellulose solubilization, and cellulose accessibility against energy, water use, and downstream enzyme/inhibitor profiles.

Plant wastes are heterogeneous mixtures of naturally occurring biopolymers whose relative abundances dictate the mechanical robustness, barrier performance, and processing behavior of derived packaging materials. The four principal constituents, cellulose, hemicellulose, lignin, and pectin, each contribute unique structural and functional attributes that can be tuned through selective sourcing or pretreatment (Table 2).

### 3.1. Main Components Bio-Waste Materials

Cellulose, the most abundant polymer in plant-derived wastes, is a linear polysaccharide that forms strong microfibrils, conferring structural rigidity and tensile strength. Agricultural residues, such as straw, typically contain 35–50% cellulose [46]. Hemicelluloses, which account for approximately 20–35% of lignocellulosic biomass, are amorphous branched polymers with numerous hydroxyl groups. Their high hydrophilicity undermines mechanical robustness by promoting moisture uptake and disrupting intermolecular cohesion. However, these same hydrophilic polysaccharides can form continuous films through extensive hydrogen-bonded networks when cast from solution. Moreover, low molecular weight fractions and bound water act as internal plasticizers, intercalating between polymer chains to lower the glass transition temperature (Tg) and enhance flexibility, despite the solid nature of the polysaccharide matrix at room temperature.

Lignin, a complex aromatic polymer, comprises roughly 15–25% of dry agricultural biomass [47]. It is inherently hydrophobic and provides rigidity and UV protection, acting as a natural binder in plant cell walls. However, excessive lignin can impart brittleness and dark coloration. Pectin, a highly branched polysaccharide, is particularly abundant in fruit wastes such as citrus peels and apple pomace, where contents may exceed 20% [25]. It exhibits excellent film-forming and adhesive properties owing to its capacity to build an extensive hydrogen-bonded network when cast from aqueous solution. Under mildly acidic conditions, protonation of carboxylate groups reduces electrostatic repulsion and promotes interchain hydrogen bonding, leading to acid-induced gelation. Likewise, in the presence of divalent cations, ionic cross-links form between carboxylate moieties on adjacent polymer chains, creating a three-dimensional gel network that imparts cohesive strength and viscoelastic behavior [47].

The lignocellulosic profile varies significantly between plant types. For example, corn stalks may contain 30% lignin, 15% cellulose, and 33% hemicellulose, whereas fruit peels such as oranges are enriched in pectin and soluble sugars, with lower fiber content. These compositional differences influence their optimal use: cellulose-rich residues such as straw are well suited for structural reinforcement in composites, while pectin-rich fruit wastes are more appropriate for film formation and active packaging applications [47].

Analyses further illustrate these trends. Carrot pomace, for example, contains approximately 28% cellulose and only 2% pectin, whereas banana peels have lower cellulose (~9%) but notable amounts of lignin (~11%) along with starches and pectin [48]. These diverse profiles enable targeted applications: cellulose enhances mechanical properties and is widely used in paper and cardboard; hemicellulose and pectin provide film-forming capability and adhesion but are water sensitive; lignin contributes stiffness and hydrophobicity.

All these biopolymers are biodegradable and non-toxic. Cellulose, hemicellulose, and pectin are recognized as edible-grade polymers, and lignin, a bio-based phenolic, offers additional functional benefits such as UV resistance [17]. Thus, plant waste represents a naturally occurring polymer blend, cellulose for strength, hemicellulose and pectin for film-forming and binding, and lignin for structural rigidity and water resistance. By quantifying the cellulose, hemicellulose, lignin, and residual extractive contents of a given plant waste feedstock, one can tailor processing parameters, such as extrusion temperature profiles for thermoplastic composites or solvent-casting conditions for films, and choose specific additives to achieve target mechanical strength, flexibility, and barrier properties in biocomposite packaging.

### 3.2. Influence of Composition on Material Performance of Lignocellulosic Composition

The specific ratios of cellulose, hemicellulose, lignin, and pectin in plant waste critically influence the mechanical and barrier properties of packaging materials derived from these sources. High cellulose content is directly associated with increased tensile strength and rigidity, as cellulose microfibrils contribute significantly to structural integrity. For instance, fibers such as flax, which are rich in cellulose, yield stronger composites compared to those containing higher levels of lignin. Nasri et al. (2023) demonstrated that in polypropylene composites, flax fibers, with higher cellulose and hemicellulose content—exhibited superior tensile modulus compared to lignin-rich alternatives [33].

Lignin, while conferring hydrophobicity and rigidity, can negatively impact flexibility and mechanical cohesion if present in excess. Its inherent hydrophobic nature disrupts hydrogen bonding among cellulose chains and can lead to pore formation when unevenly distributed. For example, studies incorporating pine cone lignin into film matrices reported increased pore formation and compromised mechanical and barrier properties with higher lignin concentrations. Nonetheless, moderate amounts of lignin can act as a reinforcing agent, enhancing stiffness and water resistance. However, levels above 15–20% often result in brittleness and processing challenges.

Hemicellulose, characterized by its low degree of polymerization and high hydroxyl group content, is highly hydrophilic. This contributes to moisture sensitivity and plasticization in packaging films. Alone, hemicellulose yields flexible films with moderate oxygen barrier performance but poor water resistance. Therefore, chemical modification or blending with more hydrophobic polymers is commonly employed to enhance its performance. Hemicellulose-rich fibers typically increase the moisture uptake of bio-composites, accelerating biodegradation but reducing dimensional stability. Partial removal of hemicellulose has been shown to improve fiber dimensional stability under humid conditions, reducing moisture-induced swelling and shrinkage of the fibers [49].

In citrus-peel pectin films, 15 wt% pectin increases elongation at the break by 30% (from 4% to 5.2%) and reduces Young’s modulus by 20%, providing ductility akin to low-density polyethylene. However, due to its solubility in water, pectin must often be cross-linked or blended with stronger polymers to maintain structural integrity. Pectin films (100 µm) exhibit oxygen permeability of 3 cm^3^·m^−2^·d^−1^·atm^−1^, comparable to EVOH, and >90% transparency at 600 nm when plasticized with 20 wt% glycerol [50].

Wax and oil content, although less emphasized, also significantly affect packaging performance. These hydrophobic substances can improve water resistance but may interfere with fiber bonding if not adequately removed during preprocessing. Broadly, cellulose and hemicellulose contribute to hydrophilicity, while lignin and natural waxes increase hydrophobicity. This balance is critical when designing plant fiber-based materials, as an untreated fiber network will absorb moisture readily, whereas fibers coated with lignin or wax exhibit reduced water uptake [51].

Therefore, the optimal composition of plant waste for packaging depends on the intended application. For rigid packaging formats like paper trays, a high cellulose content with moderate lignin is advantageous. For flexible or edible films, a formulation rich in pectin or starch with minimal lignin is preferred [52].

The monomer composition, molecular weight distribution, and degree of crystallinity of a biopolymer dictate its chain mobility and intermolecular interactions, which in turn determine barrier performance, mechanical strength, and thermal behavior in packaging applications. Cellulose imparts strength, hemicellulose and pectin contribute to flexibility and film-forming ability but increase moisture sensitivity, and lignin enhances hydrophobicity and rigidity but can reduce extensibility.

### 3.3. Pre-Treatment Methods for Modifying the Properties of Sustainable Packaging Materials

Pre-treatment processes are essential for converting raw plant waste into functional packaging materials, as they enable tuning of the material composition and enhancement of performance characteristics. These treatments, mechanical, chemical, enzymatic, or combined, aim to increase cellulose purity, remove impurities such as lignin and waxes, improve fiber bonding, and, in some cases, generate nano-structured fractions that significantly boost mechanical and barrier properties [53].

One widely used chemical pre-treatment is alkaline treatment, typically employing sodium hydroxide (NaOH). This process partially removes hemicellulose and lignin, increases the relative cellulose content, and enhances surface roughness to promote adhesion within composite matrices. Bekele et al. (2023) compared untreated, 5%, and 10% NaOH-treated sisal/enset fibers in composites [54]. The 5% NaOH-treated fibers demonstrated the best performance, improving tensile and flexural strength by approximately 5–9% and reducing water absorption. In contrast, 10% treatment led to fiber damage, while untreated fibers retained impurities. Similar enhancements were reported by Verma and Goh (2021), who observed an ~8% increase in tensile strength for alkali-treated jute fiber composites [55].

Mechanical refining and pulping represent another category of pre-treatments. These methods physically defibrillate fibers, improving their flexibility and bonding capability. Varghese et al. (2023) applied mechanical size-reduction to eggplant crop residues, unexpectedly improving the thermal stability of the resulting PVA composite films [17]. This effect was attributed to retained lignin forming covalent bonds with cellulose, thereby increasing thermal degradation temperatures [56].

Enzymatic treatments also play a crucial role, particularly in producing nanofibers [57,58]. Cellulases and related enzymes selectively hydrolyze amorphous polysaccharides, enhancing fibrillation. Ravindran et al. (2016) employed enzymatic hydrolysis followed by high-shear homogenization on cassava bagasse, yielding nanofibers that, when added at 0.65–1.3% to starch films, significantly improved mechanical properties and water resistance [59].

Advanced chemical techniques such as 2,2,6,6-tetramethylpiperidin-1-oxyl-mediated oxidation offer precise modification at the molecular level. Bascón-Villegas et al. (2021) used this method to produce nanofibrillated cellulose with surface carboxyl groups, achieving over 90% improvement in tensile strength when incorporated into PVA films at 7% loading [56].

Selective extraction of cellulose is critical for producing clear films or flexible packaging. Alkaline pulping is commonly used to isolate cellulose from straw, cocoa pod husk, and similar sources. For example, a 75:25 blend of cellulose extracted from cocoa pod husk and sugarcane bagasse fibers exhibited minimal water permeability [17]. Olawuyi et al. (2021) [57] applied an alkaline deep eutectic solvent (DES) to okra stalks, achieving up to 100% delignification. The isolated cellulose and mucilage were recombined into a polysaccharide film with enhanced oxygen barrier properties.

In another example, spent coffee grounds were subjected to a two-step solvent-based pre-treatment that removed waxes and polyphenols, yielding a cellulose-rich pulp suitable for paper formation. These pre-treatments are especially valuable for food wastes with high fat or phenolic content [17].

Pre-treatment technologies are crucial for tailoring plant waste to meet specific processing and performance criteria. Mechanical methods improve fiber dispersion and microstructural properties. Chemical pre-treatments, such as mercerization or acid treatment [54], enhance fiber-matrix compatibility and water resistance [58]. Enzymatic processes yield nanocellulose that reinforces packaging films even at low loadings. Optimizing these treatments for each type of waste remains a major research focus, as emphasized by Ravindran and Jaiswal (2016), who highlighted the pivotal role of pre-treatment in overcoming the inherent limitations of heterogeneous plant residues [59].

## 4. Processing Methods for Packaging Materials

Converting plant-derived polymers and hybrid composites into end-use packaging formats leverages both conventional and bespoke fabrication techniques. Melt-based approaches, such as extrusion, film blowing, injection molding, and thermoforming, require careful tuning of thermal profiles, screw designs, and cooling rates to accommodate the unique rheology and heat sensitivity of lignocellulosic biopolymers. Solution-based routes, including solvent casting, electrospinning, and spray coating, offer fine control over film thickness, porosity, and surface morphology but introduce solvent-recovery and scale-up considerations. Emerging additive manufacturing methods, like biopolymer 3D printing and foam-forming processes, further expand the design space for complex geometries and lightweight structures. Table 3 summarizes these processing platforms, detailing their operating parameters, material requirements, and representative packaging applications.

Different plant-based waste materials can be transformed into various sustainable packaging products. The table below summarizes common plant waste types, their key processing steps, the resulting packaging format, and relevant scientific references for each (Table 3).

### 4.1. Mechanical Consolidation

Mechanical consolidation processes enable the direct transformation of plant waste fibers or powders into functional packaging materials, typically using heat and pressure and, in some cases, minimal binder addition. Among the most established methods is pulp molding, which parallels conventional papermaking. In this process, plant fibers are dispersed in water, shaped, and subsequently dried under pressure. This technique relies on fiber entanglement and hydrogen bonding to form structurally cohesive products. For example, wheat straw pulp has been molded into trays using this method, yielding packaging with adequate mechanical integrity [59].

Mixed agro-waste pulps have also been employed to produce molded fiber packaging. Curling et al. (2017) fabricated trays from a combination of rice straw and long fibers using hot pressing, achieving mechanical properties comparable to traditional molded pulp products [34]. Hot pressing, a prevalent method for producing fiberboards and agro-waste panels, involves compressing chopped plant residues, often with a binder, at elevated temperatures to form rigid sheets or molded items. Sugarcane bagasse, due to its inherent binding capacity, is commercially used to produce disposable plates and bowls via this method. Sukyai et al. (2018) demonstrated that hot-pressing starch with 30% bagasse fiber produced a foam tray with enhanced strength, reduced porosity, and lower water absorption compared to starch-only foams [66].

Extrusion and injection molding techniques are utilized when plant waste is incorporated into biodegradable polymer matrices, PLA, PHA, into polymer blends that combine biopolymers with conventional thermoplastics. In these processes, plant-derived powders or milled fibers are homogeneously mixed with the molten polymer under heat and shear, then shaped into films or molded items through controlled extrusion or injection steps. Lima et al. (2021) incorporated up to 20% mango seed powder into PLA composites via extrusion and injection molding, achieving rigid packaging components without significantly compromising mechanical properties [67]. Similarly, Freitas et al. (2023) extruded PLA with rice straw fibers, enhancing biodegradability and reducing production costs through partial substitution of PLA with waste fibers [68].

**Figure 4:**
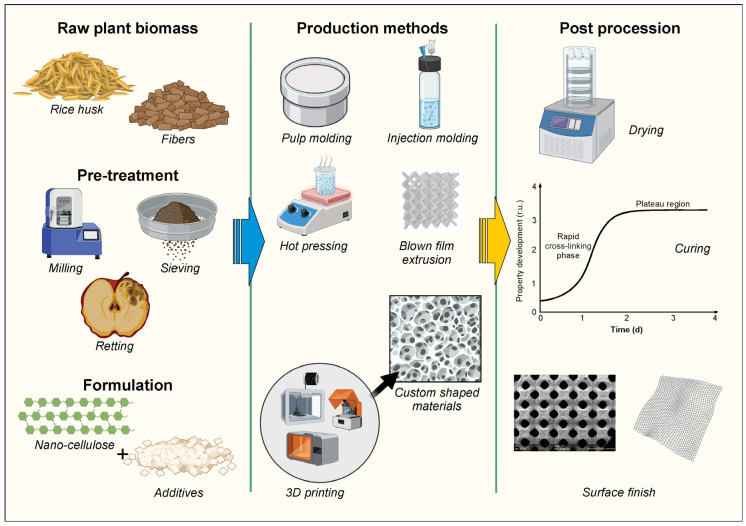
Processing methods to packaging materials. Created with BioRender.com (License No. QA28F9T0YR).

Blown film extrusion has also been adapted for plant waste use. Cristofoli et al. (2023) reported successful production of thermoplastic starch films from agricultural waste using conventional film blowing equipment, with appropriate plasticizer and formulation adjustments [37]. An emerging innovation in this space is extrusion-based 3D printing of plant-based biopolymers, where blends of nano-cellulose and biodegradable resins are printed directly into packaging geometries (Figure 4).

Mechanical consolidation methods, including pulp molding, hot pressing, and extrusion, offer effective pathways for converting plant waste into sustainable packaging. These processes are advantageous as they can leverage existing industrial infrastructure and avoid the use of harsh chemicals [69]. While minor binders are sometimes employed to enhance cohesion and water resistance, the overall approach emphasizes eco-efficiency. Ongoing research is focused on optimizing process parameters to maximize the mechanical performance and environmental benefits of these plant waste-derived materials [17].

### 4.2. Blends with Biopolymers

An effective strategy to enhance the utility of plant waste in packaging applications is to blend waste-derived materials with established biodegradable polymers. Such composites allow for the synergistic use of the unique properties of each component, optimizing mechanical strength, barrier performance, biodegradability, and cost-effectiveness. Common biopolymers used in these blends include starch, PLA, PHAs, cellulose derivatives, and chitosan.

Starch films tend to absorb water and swell because of their abundant hydroxyl groups, leading to loss of stiffness and dimensional stability under humid conditions. Reinforcing with lignocellulosic fibers creates extensive interfacial hydrogen bonds and a tortuous network within the matrix, which (a) reduces the number of free hydroxyl sites available for water binding, (b) hinders water diffusion pathways, and (c) lowers overall water uptake and swelling in high-humidity environments [70]. Berthet et al. (2015) added wheat straw fibers to a poly(3-hydroxybutyrate-co-3-hydroxyvalerate)/starch matrix to produce packaging, observing improved stiffness, though excessive fiber size increased water vapor transmission, an effect beneficial for packaging respiring produce [71].

To improve moisture resistance, fibers can be pre-treated with hydrophobic coatings [5], silane or acetylation treatments that graft alkyl chains onto fiber surfaces to repel water, and are functionalized with coupling agents (such as silane coupling agents or maleic anhydride-grafted polymers) that chemically bridge hydrophilic fibers and the starch matrix [27]. During film casting, starch gelatinizes when heated above 60–70 °C, its granules swell and leach amylose chains, which then entangle around fiber surfaces and form hydrogen-bonded interfaces. This swollen, molten starch penetrates fiber lumens and surface irregularities, promoting strong mechanical interlocking and effective fiber–matrix adhesion [37].

Nanofibers derived from cassava bagasse have also shown promise in reinforcing starch films, effectively lowering water absorption and increasing film stability [5]. These combinations are fully bio-based and often meet edible-grade standards. Starch-based products such as loose-fill packaging foams commonly include small quantities of agro-waste fibers and crosslinkers to improve foam structure and reduce material costs [27]. While hydrophobic treatments or coupling agents may be required for compatibility, gelatinization during aqueous processing typically ensures good fiber–matrix adhesion [37].

Apple-pomace powder (5 wt%) in PLA/starch bilayers reduces peroxide value in packaged meat by 35% over 7 days, demonstrating antioxidant activity alongside mechanical reinforcement. Freitas et al. (2023) combined rice straw fractions with a starch–PLA bilayer film, leveraging the antioxidant properties of the straw to extend meat shelf life, thereby adding bioactivity in addition to structural reinforcement [68].

Neat PLA films (50 µm) achieve tensile strengths of 60–70 MPa and moduli of 2000–3000 MPa, but cost USD 2500/tonne, and 30% higher than corn starch alternatives, driving blended formulations. Varghese et al. (2023) reported on PLA composites containing 20–40% rice straw, which, while reducing tensile strength and barrier properties, improved biodegradability and economic viability [17]. In PLA/rice straw blends (30 wt%), half-mass loss occurs in 60 days under industrial-composting conditions (58 °C), versus >180 days for neat PLA, while raw-material cost drops by 25%.

PHAs, including PHB, are another class of biopolymers often blended with plant waste to enhance processability and reduce brittleness. The incorporation of lignocellulosic fillers acts as a nucleating agent in the PHA melt, lowering the crystallization temperature and reducing melt viscosity; this broadens the processing window and improves melt flow during extrusion or injection molding. Sánchez-Safont et al. (2018) [72] found that, beyond thermal stabilization and modulus increases, these fillers also impart shear-thinning behavior to PHB melts, facilitating lower-temperature processing and more uniform part filling. PHB composites with rice husk have similarly shown promise in food packaging, offering satisfactory strength and improved biodegradability [35].

In some systems, plant waste-derived polymers constitute the continuous biopolymer phase, with commercial biopolymers incorporated as functional modifiers. For example, a citrus pectin–based film may be blended with PLA to enhance water resistance, while maintaining full biodegradability and food contact safety [73]. Chitosan, derived from shellfish waste, is frequently blended with plant-derived fibers to yield antimicrobial and mechanically robust packaging films. Elhussieny et al. (2020) demonstrated that incorporating rice straw nanofibers into chitosan films significantly enhanced tensile properties while preserving biodegradability [74].

Other natural polymers, such as gelatin, have also been used in combination with fruit pomace extracts to produce active packaging films with antioxidant capabilities. Generally, composite systems outperform single-component formulations, meeting food packaging criteria for strength and barrier properties, at least for certain applications.

According to a review by Cristofoli et al. (2023), pilot-scale development of such bioplastic blends is already underway [37]. The integration of agri-food by-products into biopolymer matrices not only reduces production costs but also improves performance, addressing specific limitations of individual materials [75]. Emerging commercial products, including paper–plastic hybrid packaging, exemplify how blending enhances strength, barrier function, and sustainability. The continued development of bio-based compatibilizers will further advance this field, maximizing the practical utility of plant waste in packaging.

### 4.3. Recycling of PLA and PHA-Based Packaging

Recycling of PLA and PHA biopolymers has progressed from simple composability toward integrated circular economy approaches. However, a critical appraisal reveals several technical, economic, and infrastructural hurdles that must be addressed before post-consumer PLA/PHA packaging can rival traditional plastics in recyclability. Repeated melt, reprocessing remains the simplest route yet suffers from polymer chain scission, leading to declining molecular weight, embrittlement, and compromised barrier properties after just a few cycles [76]. While chain extenders and reactive blending with amorphous PHAs show promise in laboratory settings, maintaining >90% tensile strength over multiple cycles [76], these additives introduce complexity in supply chains and raise questions about additive toxicity, regulatory approval, and cost. Moreover, blending strategies may dilute the purity of recycled streams, complicating separation and downstream reuse.

Hydrolysis and alcoholysis can reclaim high-purity monomers under mild conditions, and industrial implementations demonstrate technical viability at scale. Yet, the economic feasibility hinges on monomer market prices, energy inputs for purification, and integration with existing chemical infrastructure. Alcoholysis routes that yield solvent-grade esters require subsequent distillation steps, whose energy demands may offset environmental gains unless fueled by renewables [77]. Catalytic pyrolysis of mixed PLA/PHA waste offers a solvent-free alternative but demands precise thermal control and catalyst management to avoid unwanted byproducts and catalyst deactivation [76].

Enzymatic depolymerization leverages mild conditions to liberate lactic acid, 3-hydroxyalkanoates, and recent engineering of cutinases has cut degradation times to days at ≤50 °C with >90% conversion [78]. However, enzyme costs, stability, and product inhibition remain barriers. Microbial valorization, using recovered monomers as feedstock for new polymer biosynthesis, embodies a true biocircular loop but requires dedicated fermentation facilities and robust separation processes to isolate and purify both monomers and newly synthesized polymer [79]. Current techno-economic assessments suggest that enzyme-based recycling is promising for high-value, low-volume packaging but unlikely to compete economically for commodity packaging without significant cost reductions in enzyme production and bioreactor design.

## 5. Future Outlook

**Figure 5:**
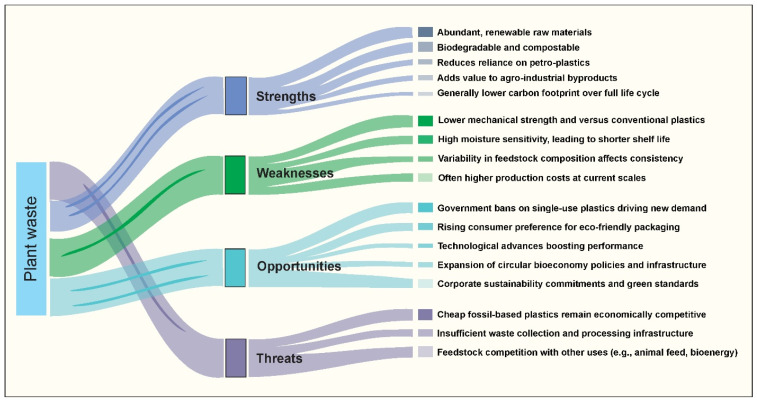
SWOT analysis of packaging materials from plant waste.

The successful commercialization of packaging derived from plant residues will be determined not only by advances in material science but also by the ability to manage highly heterogeneous biomass feedstocks, to integrate new materials into existing supply chains, and to provide supportive policy frameworks [80]. Agricultural residues differ widely in polymer composition, ash content, and moisture depending on crop species, soil conditions, and harvest season [81]. This intrinsic variability affects fiber extraction, pulping, and thermoplastic processing; reliable process control and pretreatment strategies are therefore essential to obtain consistent product quality [82]. In addition, raw biomass often lacks desirable storage and handling properties, high moisture content accelerates microbial degradation, and variability in hemicellulose complicates pulping, so supply chain logistics must be designed to minimize quality losses and deliver feedstocks with uniform particle size and composition [83] (Figure 5).

Economic feasibility remains a major barrier. Plant-waste packaging is currently produced at small scales, requiring energy-intensive pretreatments and specialized equipment; these factors often make unit costs higher than those of commodity petrochemical plastics [84]. Policy analyses emphasize that financial incentives and procurement mandates are needed to overcome cost barriers and create economies of scale [85]. Development of “green composite” formulations, in which agricultural fibers replace a portion of bioplastic resin, is also being pursued to lower material costs and valorize agro-waste [86].

Material performance is another challenge. Many starch- or protein-based films exhibit lower tensile strength and higher water vapor permeability than polyethylene or polyethylene terephthalate, limiting their use in demanding food contact applications. Research in nanocellulose-reinforced biocomposites shows that cellulose nanofibers form extensive hydrogen-bonded networks with polymer matrices, enhancing modulus, toughness and barrier properties. Incorporating 4–5 wt% cellulose nanocrystals or nanofibers into chitosan or starch films increases tensile strength by 26–39% compared with neat films, and can also improve elongation at break and thermal stability [87]. Such nano-reinforcement, combined with surface coatings or multilayer structures, offers a pathway to match or surpass the mechanical and barrier performance of petrochemical plastics. Future work should also explore compatibilizers and plasticizers that maintain flexibility without sacrificing strength [88].

End-of-life processing must be considered when claiming environmental benefits. Many plant waste-derived bioplastics are industrially compostable, but complete biodegradation (conversion to CO_2_ and water) only occurs under controlled temperatures (58 °C), aeration and moisture. A recent review notes that a large fraction of certified compostable packaging never reaches industrial composting due to inadequate collection, mis-sorting or a lack of facilities; misdirected bioplastics that end up in landfills can generate methane or fragment into microplastics, undermining sustainability claims [89,90]. The same study stresses that poor labeling and limited consumer awareness contribute to improper disposal and that ecotoxicity tests have found some bioplastic additives to inhibit microbial luminescence [91]. Expanding composting infrastructure, harmonizing labeling schemes, and ensuring chemical safety of additives are therefore critical.

### Integration into Existing Value Chains

Broad adoption of plant-waste packaging will require integration with current agricultural, industrial and waste-management systems [92]. Developing reliable supply chains is essential (Figure 5). Harvest residues such as straw, husks and bagasse are geographically dispersed and seasonal; organizing collection cooperatives and establishing dedicated logistics for drying and densification can transform these residues from waste into feedstocks [93]. In developing countries, around 60% of cellulose used in paper comes from non-wood resources such as jute, sisal, bamboo, and bagasse, illustrating that integration of agricultural fibers into the pulp and paper sector is already feasible [94]. Conventional paper mills often blend non-wood fibers with wood pulp to improve runnability and utilize surplus agricultural residues, while plastic processors increasingly modify screw designs and cooling protocols to accommodate bio-composites [95].

Integrated biorefineries represent another pathway. These facilities can fractionate lignocellulosic residues into multiple product streams—fermentable sugars for biofuels, lignin-derived resins for adhesives, and cellulose fibers for packaging—thereby improving overall economics [96]. Aligning material flows in a circular bio-economy ensures that waste from one process becomes feedstock for another, reducing dependency on virgin resources. Modular integration approaches, such as compounding plant fibers into thermoplastic pellets or retrofitting pulp-molding lines to handle agricultural fibers, allow gradual adoption with existing equipment.

End-of-life compatibility must also be engineered. Many plant-waste materials are compostable or recyclable in paper streams. Industrial composting at 58 °C can achieve >90% mass loss within 60 days, but only about half of compostable packaging currently reaches appropriate facilities [89,97]. Harmonizing material design, labeling, and collection with composting and recycling infrastructures can prevent contamination and ensure that packaging actually biodegrades or is recycled. Policy developments, such as the European Union’s 2030 requirement that all packaging be reusable, recyclable, or compostable, support this alignment. Treating agricultural residues as secondary crops can open new income streams for farmers; pilot programs that collect rice stubble for packaging production have demonstrated the feasibility of this model [98].

## 6. Conclusions

This review demonstrated that cellulose-, hemicellulose-, lignin-, pectin- and protein-rich residues from crops such as wheat straw, corn stover, sugarcane bagasse and brewers’ spent grain can be converted into films, trays and foams with competitive mechanical strength and thermal stability when properly pre-treated and blended with biopolymers like starch, PLA and PHAs. Advances in processing technology, such as twin-screw extrusion of fiber-reinforced composites, solvent-free reactive extrusion for lignin-based plastics and enzymatic or deep eutectic solvent pre-treatments to improve fiber dispersion, are critical for scaling up production; here, scaling up refers to transitioning from laboratory-scale demonstrations to pilot and industrial-scale manufacturing while maintaining material performance and process efficiency. Integration with existing papermaking and plastics processing lines requires specific modifications and co-located biorefineries can valorize multiple waste fractions into packaging materials, chemicals and biofuels. End-of-life strategies must be matched to material composition: fiber-based packaging can enter paper recycling streams, while starch- or PHA-rich composites can be industrially composted under controlled conditions. Continued materials-science research, such as improving compatibilisation between hydrophilic fibers and hydrophobic matrices and optimizing barrier properties, alongside targeted engineering improvements in processing equipment, will determine whether plant waste-based packaging can achieve commercial competitiveness and contribute significantly to a circular bioeconomy.

## Figures and Tables

**Table 1:** Sources of plant waste and their packaging applications.

Source	Composition	Packaging Application	Key properties and Benefits	Ref.
Wheat straw	Rich in cellulose (~40%), hemicellulose, lignin.	Molded pulp trays and packaging inserts	High strength comparable or superior to EPS foam (tensile modulus ~0.47 MPa for 80% straw vs. 0.16 MPa for EPS) and fully biodegradable.	[15,16]
Rice husks	High lignocellulosic fiber content (≈35–45% cellulose); also contain silica.	Reinforcement in bioplastic films	Enhanced tensile strength (~60% improvement with husk fiber addition) and reduced reliance on synthetic polymers. Often also imparts antimicrobial or antioxidant properties when treated	[17]
Sugarcane bagasse	Fibrous residue of sugar cane; ~40–50% cellulose, 25–35% hemicellulose, 20–25% lignin.	Biodegradable tableware; filler in biopolymer composites	Improves composite stiffness and lowers water permeability. Bagasse–fiber composites show reduced water vapor transmission and competitive mechanical strength, and are fully compostable in disposal.	[18,19]
Corn husks and stover	High cellulose fiber content	Pulp for paper and cardboard packaging; biodegradable paper products	Can replace wood pulp fibers in paper packaging up to ~80% without loss of strength. Corn husk fibers provide adequate mechanical integrity for paper and are renewable alternatives to wood.	[20,21]
Mixed fruit peels	Rich in pectin, starches, and polyphenolic antioxidants.	Bioplastic films and edible packaging	Intrinsically antimicrobial and antioxidant; helps extend shelf-life of fresh produce by inhibiting spoilage. For example, peel-derived films showed antimicrobial activity and prolonged fruit freshness.	[22,23]
Apple pomace and peel	High in pectin and polyphenols (antioxidants).	Active food packaging films	Provides antioxidant and antimicrobial functions. Apple peel polyphenol-infused films inhibit bacterial growth, suitable for meat or perishable foods.	[17,21]
Citrus peels	High pectin content; flavonoids and essential oils present.	Coatings and laminates for paper or bioplastic films. For instance, mandarin peel extract is used as a paper coating.	Improved barrier properties: coatings from citrus waste significantly reduced gas and water permeability and oxygen transmission of Kraft paper. Also adds antioxidant capacity to packaging.	[24]
Potato peels	Contain starch, dietary fiber, some protein, and phenolics.	Edible films and loose-fill foam packaging. Also used in biodegradable packing peanuts.	Potato peel-derived films exhibit an elastic modulus of 36 MPa, comparable to low-density polyethylene (LDPE, 25–80 MPa), demonstrating sufficient stiffness for flexible packaging. Native peel lipids and phenolic compounds reduce surface energy, yielding enhanced hydrophobicity (water contact angle > 80°). Foam articles molded from potato peel pulp cushion shocks as effectively as expanded polystyrene peanuts, yet are fully compostable under industrial conditions.	[25,26]
Sweet potato starch	Starch from sweet potato; reinforced with fibrous residues	Compostable foam trays for food packaging	Fiber reinforcement greatly improves both compressive strength and heat resistance of sweet potato starch foams. A formulation containing 5 wt% bagasse and asparagus fiber retained its cell structure up to higher temperatures, demonstrating enhanced dimensional and mechanical stability under heat, while also exhibiting lower water uptake than an unfilled starch foam.	[27]
Parsley and spinach stems	Dried herb, vegetable stems	Filler in bio-composite films	Acts as a natural filler and active additive. High loading (30–70 wt%) of micronized parsley and spinach stems in a biopolymer film provided antioxidant activity and added fiber reinforcement.	[28]
Sawdust and wood chips	Primarily cellulose (~50%) and lignin (~25%); fibrous wood pulp.	Molded fiber packaging (trays, plates, panels) as an alternative to Styrofoam.	Excellent strength-to-weight ratio and shock absorption. Pulp molded from wood, sawdust achieves comparable tensile strength to EPS foam, while being biodegradable. Often used in combination with other agro-fibers to improve sustainability and reduce cost.	[15,16]
Pine needles	Dried pine leaves; ~40% cellulose, high aromatic extractives.	Functional paper and fiber boards with active properties (e.g., ethylene-scavenging fruit packaging paper).	Pine-needle pulp provides a porous, renewable matrix for nano-zeolite impregnation. The zeolite adsorbs up to 62% of ethylene gas emitted by fruits at ambient temperature, slowing ripening and extending shelf-life. By valorizing forest residues, a wildfire fuel hazard, into a support for active ethylene scavengers, this approach creates high-value, compostable packaging.	[29]
Coconut coir fiber	High lignin (~41–45%) and moderate cellulose (~36–43%) content; very durable natural fiber.	Reinforcement in biopolymer composites (e.g., PLA or starch-based films, molded items).	Improves tensile strength and moisture resistance of composites. Coir-PLA biocomposites showed ~40% higher tensile properties than many other natural fiber composites. High lignin content imparts hydrophobicity, reducing water absorption in packaging.	[30]
Brewers’ spent grain	Mixture of fiber (cellulose ~17%, hemicellulose ~28%), protein (~20%), lignin (~12%), plus polyphenols.	Filler in biodegradable films and foils (starch, Polyvinyl alcohol (PVA), or PLA-based); also processed into paper sheets.	Films and coatings made from brewer’s spent grain (BSG) combine the grain’s residual proteins and phenolic compounds with its fibrous cellulose matrix. The protein–fiber network increases tensile strength and reduces gas permeability, while the bound phenolics provide antioxidant activity and UV-scavenging “active” functionality. This valorizes a high-volume brewery byproduct into fully compostable packaging with both barrier and active properties.	[31]
Residual coffee grounds	Lignocellulosic biomass with high lignin (~30%), cellulose (~15–20%), oils, and coffee polyphenols.	Pulped into paper sheets and molded packaging; also used as filler in bioplastics.	After solvent pre-treatment to remove oils, coffee grounds can yield cellulose-rich pulp for paper. The resulting packaging is biodegradable and can incorporate coffee’s bioactive compounds for antioxidant and antimicrobial effects. Diverts large amounts of coffee waste from landfills	[32]
Flax fiber residues	Very high cellulose content with some hemicellulose and lignin.	Reinforcement fiber in plastic composites	A 50:50 (*w*/*w*) flax-fiber/polypropylene composite, not a simple polymer blend, achieves up to 25% higher tensile strength and 15% greater flexural stiffness than neat PP under identical processing conditions. Under standardized Izod impact testing, this formulation also absorbs more energy before fracture. Such gains in impact performance require the selection of a relatively low molecular weight PP matrix and will vary with resin grade, fiber treatment, and test metric used.	[33]

**Table 2:** Chemical composition of common plant wastes for sustainable packaging applications. Note: CEL, cellulose; HO, hemicellulose; LP, lignin; PCT, pectin; STR, starch; PRO, protein.

Plant Waste	CEL, %	HO, %	LP, %	PCT, %	STR, %	PRO, %	Functional Components	Ref.
Wheat straw	32–47	19–35	5–24	5	-	3–5	1% wax (cuticular lipid)	[38]
Rice straw	30–38	19–32	7–13	2.8	-	3	trace waxes present	[38]
Sugarcane bagasse	40–50	25–35	20	-	-	1–3	minor waxes/extractives (~5% total)	[39]
Rice husk	35	25	20	-	-	3	high silica ash (~17% inorganic)	[40]
Corn stover	35–50	20–35	12–20	5	-	3	-	[41]
Banana peel	7.6–9.6	6.4–9.4	6–12	10–21	3	6–9	3.8–11% crude lipids; polyphenols 0.2–0.85% (tannic eq)	[42]
Coconut husk	32–50	0.2–15	30–46	3–4	-	-	-	[43]
Pineapple leaf fiber	80	6–12	5–12	-	-	-	-	[44]
Orange peel	22	11	2	25	-	6	-	[45]

**Table 3:** Processing methods for plant waste into packaging materials.

Plant Waste	Main Processing Steps	Packaging Format	Ref.
Sugarcane bagasse	Cleaning and mechanically pulping to obtain fibrous pulp; pulp is molded into shape and heat-dried	Molded fiber products	[60]
Wheat straw	Chemical pulping to isolate straw fibers; fiber pulp molded under pressure into products	Molded pulp packaging	[60]
Rice husk	Mechanical grinding of rice husks into fine flour; melt-blended with biopolymer and thermo-compression molded	Bioplastic composite films	[17]
Corn husk	Alkaline pretreatment and fiber extraction from corn husks; fibers mixed with plasticized starch and solution cast into films	Hybrid starch-based films	[61]
Banana pseudo-stem fiber	Chemical delignification (acid and alkali) to extract cellulose from banana stem fibers; cellulose dissolved in ionic liquid and cast into films	Pure cellulose films	[62]
Pineapple leaf fiber	Acid hydrolysis to obtain cellulose nanocrystals (CNC) from pineapple leaf waste, CNC dispersed in polymer (PVA) solution, and cast into films	Bionanocomposite films	[63]
Coconut coir fiber	Alkaline treatment of coir (coconut husk) fibers for improved adhesion; fibers extruded or compounded with bio-polymers and molded	Fiber-reinforced biocomposites	[64]
Cocoa pod husk	Alkaline pulping of cocoa pod husks to extract cellulose; cellulose (with optional added fibers) solution cast into film form	Cellulose-based films	[17]
Oil palm EFB fiber	Mechanical refining of oil palm empty fruit bunch (EFB) fibers into pulp; vacuum-assisted molding of wet pulp into designed shapes	Molded pulp trays	[65]

## Data Availability

The data presented in this study are available on request from the corresponding author.

## Abbreviations

The following abbreviations are used in this manuscript:

BSG	Brewers’ spent grain
CEL	Cellulose
CNC	Cellulose nanocrystals
DES	Deep eutectic solvent
HC	Hemicellulose
HO	Hemi-oligosaccharides
LP	Lignin phenolics
MFC	Microfibrillated cellulose
PCT	Pectin
PHA	Polyhydroxyalkanoates
PLA	Polylactic acid
PO	Pectic oligosaccharides
PVA	Polyvinyl alcohol
PRO	Protein
STR	Starch
